# Validation of a newly adapted Chinese version of the Newest Vital Sign instrument

**DOI:** 10.1371/journal.pone.0190721

**Published:** 2018-01-05

**Authors:** Jin Xue, Yongbing Liu, Kaixuan Sun, Linfeng Wu, Kai Liao, Yan Xia, Ping Hou, Huiping Xue, Hongcan Shi

**Affiliations:** 1 Medical Academy, Yangzhou University, Yangzhou, Jiangsu Province, China; 2 Guangling College, Yangzhou University, Yangzhou, Jiangsu Province, China; 3 College of Nursing, Yangzhou University, Yangzhou, Jiangsu Province, China; Eberhard-Karls-Universitat Tubingen Medizinische Fakultat, GERMANY

## Abstract

**Objective:**

To develop a Chinese version of the Newest Vital Sign (NVS-CHN) instrument and evaluate its psychometric properties.

**Methods:**

To deal with cross-cultural adaptation problems, after translation of the NVS into Chinese, the Delphi method was used for experts and cognitive testing was used for participants. A cross-sectional study including 351 participants was conducted to assess the validity of the NVS-CHN. Internal reliability, criterion validity, and known-groups validity were investigated. The NVS-CHN was further validated against a suitable standard, the Chinese Citizen Health Literacy Questionnaire (CCHLQ).

**Results:**

The validity of the NVS-CHN was established by conducting a Delphi survey (three rounds) and cognitive testing (three rounds). Cronbach’s alpha was 0.71, indicating that internal consistency was acceptable. A Spearman’s correlation coefficient of 0.68 between the NVS-CHN and CCHLQ revealed excellent criterion validity. Differences in NVS-CHN scores by education level confirmed known-groups validity. A receiver operating characteristics analysis showed that the area under the curve was 0.81, indicating that the NVS-CHN was an accurate health literacy assessment tool. A score ≥ 4 out of 6 best identified participants with adequate health literacy.

**Conclusions:**

The NVS-CHN has excellent psychometrical reliability and validity, which make it a suitable tool to evaluate health literacy in China.

## Introduction

Health literacy is the degree to which individuals have the capacity to obtain, process, and understand basic health information and services needed to make appropriate health care decisions [1]. Health literacy level is associated with several known social demographics, including education and age [2–4]. People with higher education tend to have higher heath literacy. A positive correlation has been observed between education level and health literacy, while the correlation for age is the opposite direction, as older people have a lower level of health literacy. People with low health literacy are generally lack of self-management skills, have poor health outcomes, use more emergency resources, show poor understanding of health literacy, and have a high risk of mortality and morbidity in older adults [5–9].

In light of the close correlation between health literacy and health outcomes, assessing health literacy is very important. In China, the only well-established health literacy assessment tool is the Chinese Citizen Health Literacy Questionnaire (CCHLQ), developed by the Chinese Health Education Center [10]. The CCHLQ extensively tests existing health knowledge, beliefs, and skills. However, the tedious content makes it unpractical and time consuming. It is also inefficient, which makes health literacy research work laborious. Therefore, a new health literacy assessment tool is needed in China.

An accurate and quick health literacy assessment tool is needed. The Newest Vital Sign (NVS) is one of the most widely used international health literacy assessment tools and meets these requirements well [11]. It was developed in the US and had been validated in several countries including England, Netherlands, Japan and some other countries [12–15]. The NVS consists of an ice cream label and six associated questions testing numeracy and reading ability. The tool only takes about 3 minutes to administer [16] and has been used for health literacy assessments in a variety of patients and residents with different sociodemographic backgrounds [17–19].

The objectives of this study were to develop and validate a new version of the NVS (NVS-CHN) based on Chinese background. The study consisted of two phases: (1) development of the NVS-CHN; and (2) assessment of the reliability and validity of the NVS-CHN.

## Methods

### Ethical considerations

The Ethics Committee of the Yangzhou University Medical Academy approved the study. Participants were informed about the purpose of the study, and written informed consent was obtained for all study participants prior to participation.

### Phase 1: Development of the NVS-CHN

#### Translation and cross-cultural adjustment of the original NVS

The translation and cross-cultural adaptation of the original NVS proceeded via several steps according to established guidelines [20–22]. A forward translation of the original NVS into the target language (Chinese) was performed independently by two native Chinese physicians who were proficient in English. A third Chinese translator compared the two translations and synthesized them into a single Chinese version. Two additional English translators, who were blinded to the original NVS, had a medical background and were fluent in Chinese, developed the backward translation. This back translation showed no discrepancies with the original NVS.

Subsequently, we carried out the cultural adaptation using the Delphi method; this was a critical phase of the study [23]. The cultural adaptation was performed according to three principles: (1) the food products chosen should be familiar to Chinese people; (2) the nutrition labels should conform to the rules for Chinese nutrition labeling; and (3) the new version of the NVS should be comparable to the original. A panel of 25 experts, including specialists in health literacy research, nutrition management, statistics, education, public health, and clinical practice, was invited to participate in the web-based Delphi study. According to established principles, they were asked to compare the original nutrition label with the Chinese label, and provide suggestions for modifications of the information contained in the original label to ensure utility as a Chinese food label. The experts then rated (on 5-point Likert scales) the applicability of the content and layout of the proposed NVS-CHN and the equivalence between the original and modified versions of the instrument. Scores ranged from 1 to 5, with higher scores indicating greater applicability of the NVS-CHN or a higher degree of equivalence between the original and modified version of the instrument. We made further revisions to the NVS-CHN according to the suggestions and scores of the experts. The assessment procedures for the web-based Delphi survey were repeated several times until all experts gave scores of 4 or 5 and there was no disagreement.

#### Cognitive testing for further refinement of the NVS-CHN

We explored whether there were issues regarding understanding and acceptance of the proposed NVS-CHN via one-on-one interviews with the general public. Public places were chosen randomly, such as shopping malls and train stations in Yangzhou, Jiangsu Province, China. All participants were ≥ 18 years of age and Chinese speakers. We recruited participants from diverse demographic backgrounds, including people of different age and education level. Participants were asked to explain their answers in detail while completing the proposed NVS-CHN. After completing the instrument, we asked the participants to comment on the layout and wording of the labels. Each round of interviews included 20 participants. After each round, modifications were made in response to participant feedback, and the rounds continued until no more new information was gleaned. A total of three rounds of cognitive tests were performed.

### Phase 2: Assessment of the validity and reliability of the NVS-CHN

#### Sample and recruitment

At this stage, participants were also recruited from among the public places: we recruited adults among whom at least 30% were highly likely to have low health literacy, such as people with a lower education level (junior high and below) and older people. Participants were adults ≥ 18 years of age who had the ability to read and were willing to cooperate with the investigators. People with a visual impairment, no education (inability to read), cognitive impairment (inability to understand) or a healthcare occupational background were excluded.

#### Measurements

The CCHLQ, an acceptable reference standard for health literacy assessment, covers three dimensions of knowledge and belief literacy, behavior literacy and skill literacy with 15 items of true or false questions, 65 items of multiple-choice questions [10]. The score for true or false questions is one point and the score for multiple-choice questions is one or two points, making the maximum number of points 100. According to the CCHLQ score, health literacy can be divided into two categories: inadequate (0–79) and adequate (80–100). Participants were asked to complete the questionnaire themselves. As participants with primary school education were expected to generally have difficulty completing the questionnaire independently, given its tedious nature, and because their compliance was low, the questionnaire was finished via face-to-face interviews for that group. The NVS-CHN was administered during one-on-one interactions between an investigator and a participant. The participants were asked to complete a sociodemographic characteristics table before they started filling out the questionnaire.

### Statistical analyses

The Kolmogorov–Smirnov test was performed to test the normality of the distributions: the research data were non-normal distribution. Descriptive statistics were calculated for the NVS-CHN and CCHLQ scores. To assess known-groups validity, possible subgroup differences in health literacy scores, by education level and age, were examined with the Kruskal-Wallis test. Internal reliability was measured by Cronbach’s alpha coefficient. Criterion validity of the NVS-CHN was assessed the correlation (Spearman’s r) between scores on the NVS-CHN and the CCHLQ-the reference standard.

The area under the curve (AUC) was calculated from the receiver operating characteristic (ROC) curve using the CCHLQ. Youden’s index was calculated for the different NVS-CHN cut-off scores to determine the degree of health literacy.

Data were analyzed using the Statistical Package for the Social Science (SPSS version 23.0) software.

## Results

### Phase 1: Development results of the NVS-CHN

After three rounds of evaluation, all of the experts reached a consensus regarding the feasibility of the NVS-CHN and believed that was comparable to international versions, and that its degree of intelligibility for Chinese respondents was satisfactory. However, some modifications and changes were made. For example, we selected “ice cream cone” as the term for one nutritional label because this food product was popular and had similar packaging to that displayed in the original NVS. Furthermore, the “1/2 cup” serving size was replaced by “100 g”, which is widely used in China. Six of the questions were the same as those used in the original NVS. Scores on each question yielded 1 point, giving a maximum possible score of 6. In order to explore whether they understood the reason of allergy, one supplementary question to Q6 (question 6) would be used in people who answered ambiguously to the Q6 of why it was unsafe to eat the ice cream. The participants would get one score when Q6 and the supplementary questions were answered correctly.

In total, 60 participants were recruited to perform the cognitive test (20 participants/round). Problems (mostly reported by senior citizens with less education) were solved by adjusting the layout. A picture of an ice cream cone was attached to the instrument to make that nutritional label easier to understand. The final version of the NVS-CHN was then established.

### Phase 2: Results validation

#### Participant characteristics

In total, 351 participants (51.3% males) were recruited to the validation study. The median age (interquartile range, IQR) was 41 (27–54). Regarding education level, primary school and below accounted for 8.0% of the sample, while junior high school accounted for 23.6%, senior high school and technical second school accounted for 26.5%, and college and above accounted for 41.9%.

Taking into consideration that the use of health services increases with age, a specific subgroup analysis in older patients is necessary. Aged 55 and over is the inclusion criteria in many health literacy researches of the elderly [24–26]. Among the 351 participants, 76 were ≥ 55 years of age (56.6% males). The median age (IQR) was 64.5 (60–73). Regarding education level, primary school and below accounted for 14.5% of the sample, junior high school accounted for 22.4%, senior high school and technical second school accounted for 38.2%, and college and above accounted for 25.0%.

#### Psychometric properties of the NVS-CHN

As shown in Table 1, NVS-CHN scores decreased significantly with increasing age (P<0.001).

**Table 1:** NVS-CHN score comparison in different age groups (Median [IQR]).

	N(%)	NVS-CHN	P-value
Age			0.000
18–24	61(17.4%)	5(4–6)	
25–34	67(19.1%)	5(3–6)	
35–44	73(20.8%)	3(2–4)	
45–54	74(21.1%)	3(2–4)	
55–64	38(10.8%)	3(1–4)	
>65	38(10.8%)	2(1–3)	

A significant difference in NVS-CHN scores was observed among the education level groups, regardless of age (Table 2). A higher education level was associated with a higher mean NVS-CHN score (P<0.001).

**Table 2:** NVS-CHN score comparison in different education level (Median [IQR]).

	age≥18(N = 351)			age≥55(N = 76)		
Education	N(%)	NVS-CHN	P-value	N(%)	NVS-CHN	P-value
Primary school or below	28(8.0%)	1(0–1)	0.000	11(14.5%)	1(0–2)	0.000
Junior high school	83(23.6%)	3(1–4)		17(22.4%)	1(1–2)	
Senior high school and technical second school	93(26.5%)	3(3–5)		29(38.2%)	3(2–4)	
Graduate and above	147(41.9%)	5(4–6)		19(25.0%)	4(3–5)	

The number of participants that answered correctly only one question of NVS-CHN was distributed as follows: 12 for Q1, 0 for Q2, 2 for Q3, 5 for Q4, 23 for Q5, and 0 for Q6. Besides, among the participants that only failed one question, the failed question was distributed as follows: 2 for Q1, 6 for Q2, 13 for Q3, 5 for Q4, 0 for Q5, and 31 for Q6. The accuracy rates of six questions were as follows: 74.64% for Q1, 68.95% for Q2, 51.28% for Q3, 64.39% for Q4, 63.53% for Q5, 30.77% for Q6. Only 28.9% participants obtained a NVS-CHN score ≥ 4, while 11.8% scored 0, and 21.1% scored 1.

The correlation between NVS-CHN and the CCHLQ scores was significant among the sample of 351 participants (r = 0.68, P < 0.001) and remained significant in the older age group alone(r = 0.77, P < 0.001). Both of these results indicated excellent criterion-related validity of the NVS-CHN. Furthermore, NVS-CHN scores were significantly correlated with education level (r = 0.55, P < 0.001) and age (r = -0.45, P < 0.001) among all participants. CCHLQ score ranges from 43 to 93. Few participants got a CCHLQ score of more than 90, while 17.7% participants obtained a highest NVS-CHN score of 6. The internal consistency of the NVS-CHN was acceptable (Cronbach’s Alpha = 0.71) [27].

**Fig 1:**
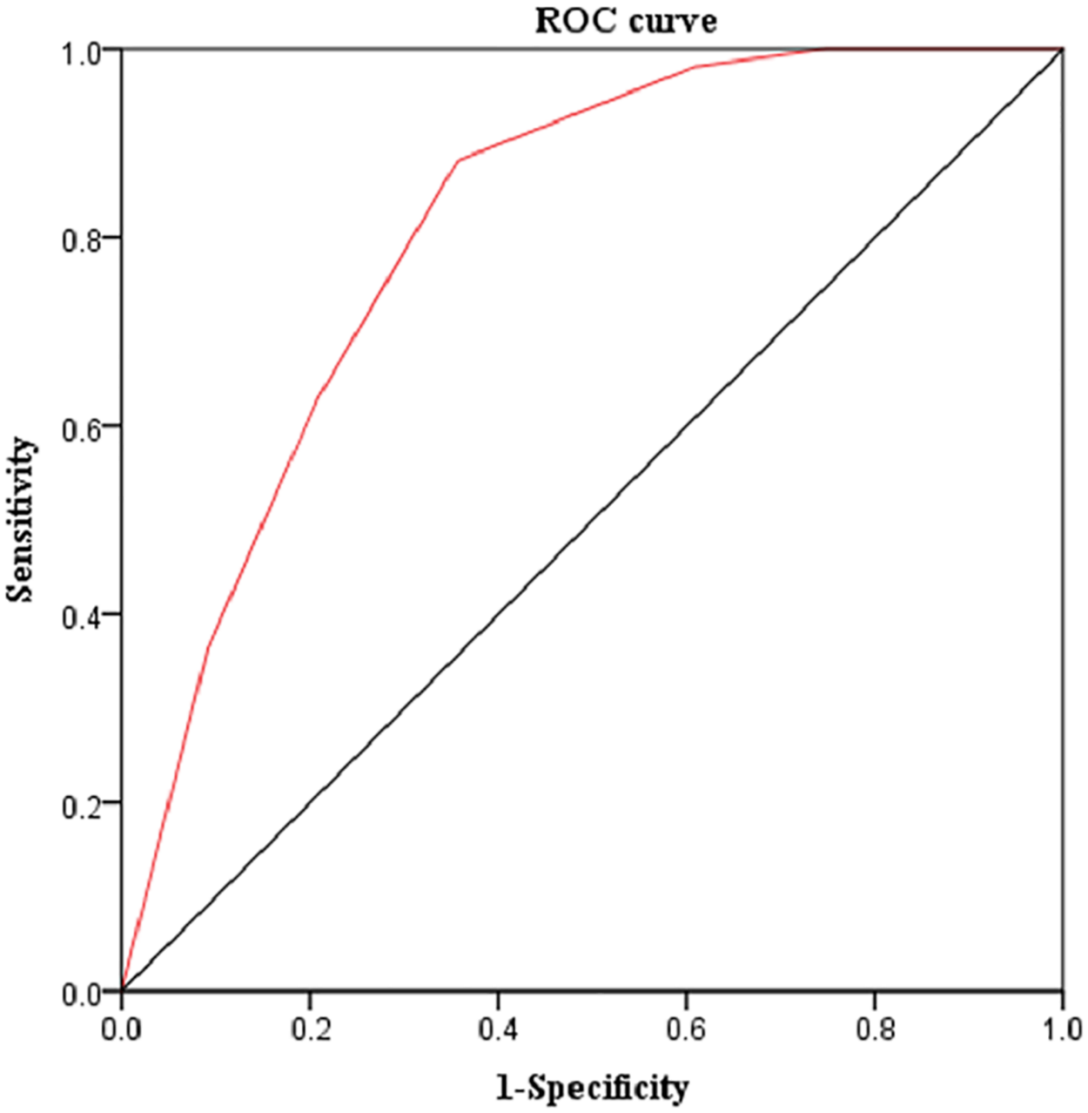
Receiver operating characteristics curve: Abilities of NVS-CHN to predict CCHLQ scores.

As shown in Fig 1, the AUC for predicting adequate health literacy determined by the CCHLQ was 0.81. The ROC analyses explored sensitivity and specificity for different NVS-CHN score cut-off points, which were established as an index of adequate health literacy as defined by the CCHLQ (Table 3). The sensitivity of the analysis of adequate health literacy decreased as the threshold level increased, but specificity increased. The optimal cutoff value for the NVS-CHN was 4, with a highest Youden’s index of 0.52. The NVS-CHN cut-off level of ≥ 4 correctly identified 88% of the participants as having adequate health literacy (CCHLQ ≥ 80), and 64% as having inadequate or marginal health literacy (CCHLQ < 80).

**Table 3:** Optimal cut-off points for prediction of adequate health literacy.

Cut point	Sensitivity(true positive)	Specificity(true negative)
6	0.36	0.91
≥5	0.63	0.79
≥4	0.88	0.64
≥3	0.98	0.39
≥2	1	0.25
≥1	1	0.08
0	1	0

## Discussion

### Principal findings and results in context

We have developed a new version of the NVS which is appropriate for performing in China. The NVS-CHN correlated well with the reference standard-CCHLQ both in the 351 participants scores and the 76 elderly participants scores, which indicated that the NVS-CHN had excellent criterion validity. The mean scores of NVS-CHN were significantly higher for those with a relatively higher level of education than for those with lower education level. It showed excellent known-groups validity of the NVS-CHN. Furthermore, the older the participants, the lower mean scores they were more likely to get. The internal consistency of the NVS-CHN was acceptable. All these indexes showed excellent psychometric properties of the NVS-CHN.

Moreover, the AUC value of 0.81 was high and appropriate NVS-CHN cut-off values for health literacy level were established. The highest Youden’s index value suggested that a score of ≥ 4 corresponds to adequate health literacy. This cut-off score for predicting adequate health literacy is the same as that for the original NVS and the subsequently developed British and Dutch versions [11–13]. Percentages of the participants in the highest score ranges between the NVS-CHN and CCHLQ group showed difference (17.7% vs 0.3%). The reason may be that CCHLQ is more difficult compared with NVS, and even medical workers who have high health literacy cannot get full marks of the CCHLQ.

Simplicity, accuracy, and rapidity are an trend in the development of a new health literacy assessment tool. The NVS-CHN is a short, time-efficient and objective assessment tool with good reliability and validity. All of these properties make the NVS-CHN an ideal tool for health literacy assessments. Notably, a study by Singh found that many participants did not fully understand the nutrition label for ice cream [28]; the same problem existed in our study. We tried to make this nutrition label concrete and visualizable. A color picture of ice cream cones was shown to participants. The responses from the participants indicated that this method was effective. Some studies have reported a floor effect in the use of the NVS in older adults [29–30]. However, our data did not show this effect. This may be because we used a colorful picture to improve the readability of the ice cream nutrition label, thus promoting greater understanding of the content among the older participants. It can reduce the floor effect in the use of the NVS in older adults, to some degree. The second reason may be that 63.1% of the older participants who we recruited had an education level of high school and above. The applicability of the NVS to older populations still needs further study.

Q5 (“Is it safe for you to eat this ice cream?”) on the NVS-CHN is the only item scored dichotomously, such that there is a 50% probability of selecting the correct answer by chance. Q6 concerns about the reason the participants choosing the negative answer. The accuracy rates for Q5 and Q6 were 63.53% and 30.77%, respectively, indicating that if participants answered Q5 correctly, this did not necessarily mean that they actually understood it. Among the participants who obtained one point of the NVS-CHN, 54.76% of participants scored on the Q5. In addition, among the participants who obtained 5 points of the NVS-CHN, no participants lost on the Q5. All of these results demonstrated that, as happened in previous versions, Q5 was correct by pure chance in many instances. Hence, NVS-CHN overestimates the healthy literacy. Salgado suggested that Q5 and Q6 be scored as a composite question [29]. However, there is no such attempt and research currently and almost all studies have scored Q5 and Q6 separately [12–15, 31]. Furthermore, health literacy is a multidimensional concept comprised of different components. This involves psycho-cognitive judgments and decision-making in health literacy [32]. Therefore, whether Q5 and Q6 of the NVS-CHN are needed to be merged is still needs further study.

### Limitations

Some limitations of this study should be considered. First, there may have been bias in the selection of participants. People who seemed friendly or easily approachable may have been more likely to be asked to participate. Second, whether NVS-CHN scores predicted health outcomes was not assessed, thus, a future study is needed to investigate the relationship between NVS-CHN scores and health outcomes.

## Conclusions

Here, we have discussed the NVS, an international health literacy tool, and validated a new version, the NVS-CHN, for use in mainland Chinese populations. We believe that the NVS-CHN has good reliability and validity and is suitable for use in Chinese populations.

## Supporting information

S1 Table Social demographic characteristics of participants in the cognitive test of the NVS-CHN. (DOCX)

S1 Data Dataset file. (XLSX)
