# Supplementary material for: Validation of a newly adapted Chinese version of the Newest Vital Sign instrument
Source: PLoS One. 2018 Jan 5;13(1):e0190721. doi: 10.1371/journal.pone.0190721 (PMC5755884; doi:10.1371/journal.pone.0190721)
Supplement: S1 Table — (DOCX) [file pone.0190721.s001.docx]

**Social demographics of participants in the cognitive test of the NVS-CHN**

|  |  | N | Percentage % |
| --- | --- | --- | --- |
| Gender | Male | 32 | 53% |
|  | Female | 28 | 47% |
| Age | 18-24 | 7 | 12% |
|  | 25-34 | 12 | 20% |
|  | 35-44 | 11 | 18% |
|  | 45-54 | 13 | 22% |
|  | 55-64 | 10 | 17% |
|  | 65- | 7 | 11% |
| Education | Primary school or below | 10 | 16% |
|  | Junior high School | 12 | 20% |
|  | Senior High School and technical second school | 16 | 27% |
|  | Graduate and above | 22 | 37% |
